# Transgene Expression and Host Cell Responses to Replication-Defective, Single-Cycle, and Replication-Competent Adenovirus Vectors

**DOI:** 10.3390/genes8020079

**Published:** 2017-02-18

**Authors:** Catherine M. Crosby, Michael A. Barry

**Affiliations:** 1 Virology and Gene Therapy Graduate Program, Mayo Graduate School, Rochester, MN 55905, USA; ccrosby@lji.org; 2 La Jolla Institute for Allergy and Immunology, La Jolla, CA 92037, USA; 3 Department of Medicine, Division of Infectious Diseases, Mayo Clinic, Rochester, MN 55905, USA; 4 Department of Immunology, Mayo Clinic, Rochester, MN 55905, USA; 5 Department of Molecular Medicine, Mayo Clinic, Rochester, MN 55905, USA

**Keywords:** adenovirus, replication-competent, replication-defective, vaccines, cancer therapy, genome replication, animal models

## Abstract

Most adenovirus (Ad) vectors are E1 gene deleted replication defective (RD-Ad) vectors that deliver one transgene to the cell and all expression is based on that one gene. In contrast, E1-intact replication-competent Ad (RC-Ad) vectors replicate their DNA and their transgenes up to 10,000-fold, amplifying transgene expression markedly higher than RD-Ad vectors. While RC-Ad are more potent, they run the real risk of causing adenovirus infections in vector recipients and those that administer them. To gain the benefits of transgene amplification, but avoid the risk of Ad infections, we developed “single cycle” Ad (SC-Ad) vectors. SC-Ads amplify transgene expression and generated markedly stronger and more persistent immune responses than RD-Ad as expected. However, they also unexpectedly generated stronger immune responses than RC-Ad vectors. To explore the basis of this potency here, we compared gene expression and the cellular responses to infection to these vectors in vitro and in vivo. In vitro, in primary human lung epithelial cells, SC- and RC-Ad amplified their genomes more than 400-fold relative to RD-Ad with higher replication by SC-Ad. This replication translated into higher green fluorescent protein (GFP) expression for 48 h by SC- and RC-Ad than by RD-Ad. In vitro, in the absence of an immune system, RD-Ad expression became higher by 72 h coincident with cell death mediated by SC- and RC-Ad and release of transgene product from the dying cells. When the vectors were compared in human THP-1 Lucia- interferon-stimulated gene (ISG) cells, which are a human monocyte cell line that have been modified to quantify ISG activity, RC-Ad6 provoked significantly stronger ISG responses than RD- or SC-Ad. In mice, intravenous or intranasal injection produced up to 100-fold genome replication. Under these in vivo conditions in the presence of the immune system, luciferase expression by RC and SC-Ad was markedly higher than that by RD-Ad. In immunodeficient mice, SC-Ad drove stronger luciferase expression than RC- or RD-Ad. These data demonstrate better transgene expression by SC- and RC-Ad in vitro and in vivo than RD-Ad. This higher expression by the replicating vectors results in a peak of expression within 1 to 2 days followed by cell death of infected cells and release of transgene products. While SC- and RC-Ad expression were similar in mice and in Syrian hamsters, RC-Ad provoked much stronger ISG induction which may explain in part SC-Ad′s ability to generate stronger and more persistent immune responses than RC-Ad in Ad permissive hamsters.

## 1. Introduction

Recombinant human adenoviral (Ad) vectors have shown promise as oncolytic and vaccine platforms due to their high titer production, robust gene expression, and potent immune response induction (reviewed in [1]). To date, the majority of studies in animals and in humans utilize E1-deleted Ad vectors. E1 deletion inactivates viral DNA replication and viral progeny virus replication, so these vectors are referred to as replication-defective Ads (RD-Ads). E1 is deleted primarily to increase safety by preventing the ability of the vector to cause adenovirus infections as a side effect of vector use.

While E1 deletion renders RD-Ads relatively safe, these vectors have been less effective when scaled up from mice to large animals and humans. This is due in part to the fact that when an incoming Ad delivers one genome to a cell, it is unable to replicate this DNA to amplify the transgene. In contrast, an E1-intact replication-competent Ad (RC-Ad) can replicate its genome up to 10,000 fold after delivering one genome [2]. Amplification of the transgene translates into proportionate amplification of transgene protein production in each cell. Given this, there is renewed interest in the use of Ad vectors that can replicate their genome, because their ability to amplify transgene expression could enhance transgene-directed immune responses and increase efficacy in vaccines and in cancer therapies [3,4,5,6].

One safety caveat of RC-Ads is that these vectors also produce progeny infectious viruses. In theory, this can be beneficial, since transgene amplification can be further amplified in other cells. However, this can also be dangerous, since a fully replication-competent Ad can also cause legitimate adenovirus infections. This means an RC-Ad vaccine or cancer therapy could spread to health care workers or family members. This could also create life-threatening infections in the patients themselves, particularly in the very young, the very old, the immunocompromised individuals, and cancer patients whose immune systems have been damaged by the disease or prior therapy.

**Figure 1:**
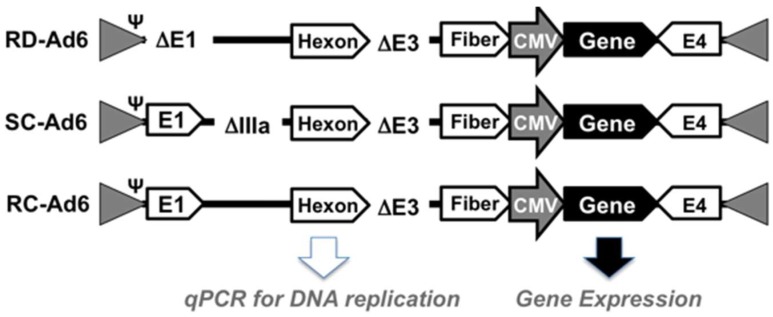
Cartoon showing adenovirus 6 (Ad6) vectors tested.

To harness the ability to replicate transgenes without the risks associated with fully replication competent Ad vectors, we recently developed single-cycle Ads (SC-Ads) by deleting key late genes rather than E1 [6,7]. The best SC-Ad currently is rendered single cycle by deletion of the pIIIA cement protein (Figure 1). This virus replicates its DNA and transgenes as well as RC-Ad, but does not produce infectious progeny viruses [6,7].

We predicted that SC- and RC-Ad could be more potent than RD-Ad given their ability to amplify transgene, despite the fact that SC- and RC-Ad both kill infected cells at the end of their viral life cycle. In contrast, RD-Ad does not itself kill cells in vitro, but these cells are ultimately executed in vivo when the immune system detects their viral antigens. It was therefore possible that the high intensity “burst” of antigen production by SC- and RC-Ad vectors might or might not out-compete RD-Ad whose lower, but less lethal expression might allow antigen expression to “integrate” longer to produce better immune responses.

When the three vectors were compared head to head in Ad-permissive Syrian hamsters, as expected, both RC- and SC-Ad vectors mediated stronger antigen expression than RD-Ad [7]. However, only SC-Ad generated stronger immune responses against transgene antigen. While this was expected for RD-Ad, we were surprised that SC-Ad was also better than RC-Ad considering that RC-Ad had the possibility for a second cycle (or more) of infection and transgene expression. This observation is not unique to single-cycle Ad, but has also been observed when comparing single-cycle and replication-competent flavivirus vectors [8].

To better understand how the vectors function as gene expression platforms, we examined here the interplay between transgene expression, the cellular reaction to infection, and cell death mediated by RD-, SC-, and RC-Ad vectors in vitro and in vivo.

## 2. Materials and Methods

### 2.1. Cell Culture

Two hundred and ninty-three human embryonic kidney cells were purchased from Microbix (Toronto, ON, Canada). A549 lung carcinoma were purchased from American Type Culture Collection (ATCC, Manassas, VA, USA). THP-1 ISG Lucia^tm^ cells were purchased from Invivogen (San Diego, CA, USA). These cell lines were maintained in Dulbecco′s Modified Eagle Medium supplemented with 10% fetal bovine serum (FBS; HyClone, Rockford, IL, USA) and penicillin/streptomycin at 100 U/mL (Life Technologies, Grand Island, NY, USA). Human small airway epithelial cells (SAECs) were purchased from Lifeline Cell Technology (Frederick, MD, USA) and maintained in BronchiaLife^tm^ media (Lifeline).

### 2.2. Adenoviruses

RD-, SC-, and RC-Ad6-GL and HA viruses were generated as described in [6,9] and shown in Figure 1. Each virus has its E3 gene cassette deleted and express a green fluorescent protein-luciferase (GL) fusion protein or influenza hemagglutinin (HA) (Figure 1A). RD- and RC-Ad6 viruses were rescued in 293 cells and purified by double CsCl banding. SC-Ad6 was rescued in 293-IIIA cells that express the IIIA protein in trans [6]. Each virus was desalted in 10% sucrose/PBS. Virus particle (vp) concentration was determined by OD260. Infectious unit (IU) were determined by Adenovirus Rapid Titer Assay (Invitrogen, Carlsbad, CA, USA on 293-IIIA cells. VP/IU ratios from [7,9] were: RD-Ad6-GL: 28, SC-Ad6-GL: 64, RC-Ad6-GL: 22, RD-Ad6-PR: 228 and SC-Ad6-HA: 174 VP/IU. Virus comparisons were performed based on virus particles rather than infectious units as recommended in [10].

### 2.3. In Vitro Luciferase Assay

1 × 10^3^ A549 and THP-1 Lucia cells were plated in black walled 96-well plates with clear well beds and these were infected a day later with 1000 virus particles (vp)/cell. At various time points, Bright Glow^tm^ luciferase reagent (Promega, Madison, WI, USA) was used was added at a 1:1 ratio and relative luminescence units was measured with the Beckman Coulter DTX 880 Multimode Detector system (Beckman Coulter, Carlsbad, CA, USA) to detect firefly luciferase. To detect secreted Lucia luciferase, QuantiLuc (Invivogen) was added instead to the wells and luciferase activity was measured.

### 2.4. In Vitro Vector Genome Quantification

A quantity of 3 × 10^5^ A549 cells were plated in six well plates, and infected at 1000 vp/cell. Two hours after infection, 1 mL of trypsin was added to cells and the cells were harvested by addition of 5 mL D-MEM with 5% FBS. Cells were centrifuges for 5 min at 300× *g* and were resuspended in 2 mL DMEM with 5% FBS. DNA was isolated from half of each sample and the remaining half was plated in 6 well plates. After 24 h, cells were harvested and DNA was isolated. DNA was isolated using the DNeasy Blood and Tissue kit (Qiagen, Hilden, Germany) according to the manufacturer′s protocol with RNase A digestion.

### 2.5. Quantitative Real Time PCR (qPCR)

Vector genomes were quantified using qPCR with primers against adenovirus hexon as in [6]. DNA sample concentrations were determined by OD260 and each was diluted to 20 ng/μL. Real-time PCR was performed on the DNA using an Applied Biosystems Prism 7900HT sequence detection system with SDS 2.3 software (Applied biosystems, Foster City, CA, USA). Each well contained 10 μL Sybr Green (Applied Biosystems, Warrington, UK), 3.8 μL H_2_O, 0.6 μL of 10 μM hexon F Primer, 0.6 μL of 10 μM hexon R Primer, and 5 μL sample (i.e., 20 ng DNA/well).

### 2.6. Animals

Inbred BALB/c mice were purchased from Harlan Sprague-Dawley (Indianapolis, IN, USA). They were housed in the Mayo Clinic Animal Facility under the Association for Assessment and Accreditation of Laboratory Animal Care (AALAC) guidelines with animal use protocols approved by the Mayo Clinic Animal Use and Care Committee. All animal experiments were carried out according to the provisions of the Animal Welfare Act, PHS Animal Welfare Policy, the principles of the NIH Guide for the Care and Use of Laboratory Animals, and the policies and procedures of Mayo Clinic (A10512).

### 2.7. Adenovirus Administration

All viruses were diluted in PBS prior to injection. For intranasal (i.n.) delivery, 1 × 10^10^ vp were diluted to 20 μL total and 10 μL was delivered per nare. For the intravenous (i.v.) route, 3 × 10^10^ vp were diluted to 100 μL and was administered by tail vein injection.

### 2.8. In Vivo Bioluminescence Imaging

Mice were anesthetized with ketamine/xylazine and injected intraperitoneally with 150 μL d-luciferin (20 mg/mL; Molecular Imaging Products, Bend, OR, USA). Animals were imaged on the Lumazone Imagine System (Photometrics, Roper Scientific, Tucson, AZ, USA) for 3 (i.v.) or 10 min (i.n.) with 1 × 1 (i.v.) or 3 × 3 (i.v.) pixel binning using no filters or photomultiplication. Lumazone imaging software (Roper Scientific, München, Germany) was used to determine luciferase activity as measured in photons/second for data analysis.

### 2.9. In Vivo Vector Genome Quantification

Female BALB/c mice were injected with the indicated vectors. 24 h later liver or lung samples were harvested and DNA was extracted using the Maxwell 16 tissue DNA purification system (Promega, Madison, WI, USA). Vector genomes were quantified using qPCR with primers against adenovirus hexon as described above.

### 2.10. Data Analysis

Graphs and statistical analyses were performed using Prism 6 Graphical software (GraphPad, La Jolla, CA, USA).

## 3. Results

### 3.1. DNA Replication and Transgene Expression in Primary Human Lung Cells

In our previous work characterizing single cycle adenoviruses, we have shown that increases in green fluorescent protein (GFP) and luciferase expression directly correlate with the ability of the virus to replicate its genome both in vitro and in vivo. Compared to RD-Ad, this translates into more robust protein expression by RC- and SC-Ad, but increased immune responses against transgene protein only by SC-Ad, but not by RC-Ad. This surprising result suggests there are differences in how SC-Ad and RC-Ad produce antigen and/or how they interact with the immune system.

In most of this work in vitro, DNA replication and transgene expression was tested in a range of immortalized human and animal cell lines whose biologies are likely perturbed to allow them to proliferate indefinitely. Cells perpetually in cell cycle and frequently in the S phase of DNA synthesis are likely to support viral infection, expression, and DNA replication better than primary cells and terminally differentiated cells. To better examine the interactions between the different vectors and cells, we tested RD-, SC-, and RC-Ad infections in non-immortal primary human small airway epithelial cells (SAECs) and in vivo in mice.

**Figure 2:**
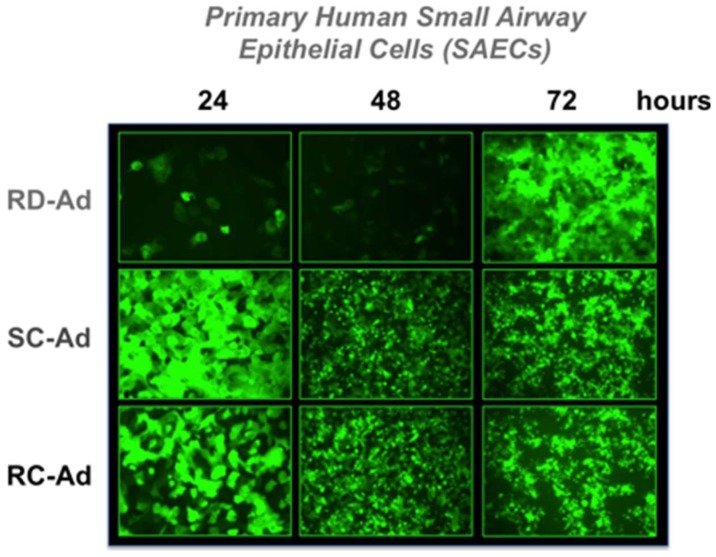
Green fluorescent protein (GFP) expression after infection of primary human lung cells with different Ad6 vectors. Cells were infected at 100 vp/cell GFP expression was visualized by fluorescence microscopy (200× at 24 h, 100× at 48 and 72 h).

**Figure 3:**
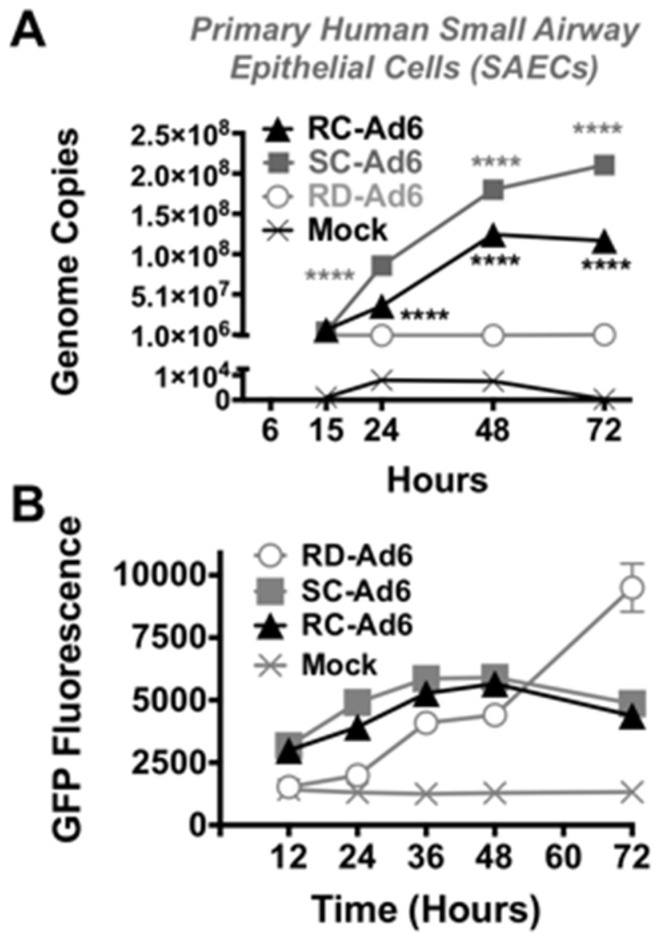
DNA replication and green fluorescent protein (GFP) expression after infection with different Ad6 vectors. Cells were infected at 100 vp/cell and viral genomes were quantified by qPCR of cellular DNA at the indicated time points (**A**). **** indicates *p* < 0.0001 by two ANOVA (analysis of variance) for single cycle (SC)- and replication-competent (RC)-Ad when compared to RD-Ad and for SC-Ad vs. RC-Ad. GFP expression was measured by fluorescence measurement on a plate reader at the indicated time points (**B**).

**Figure 4:**
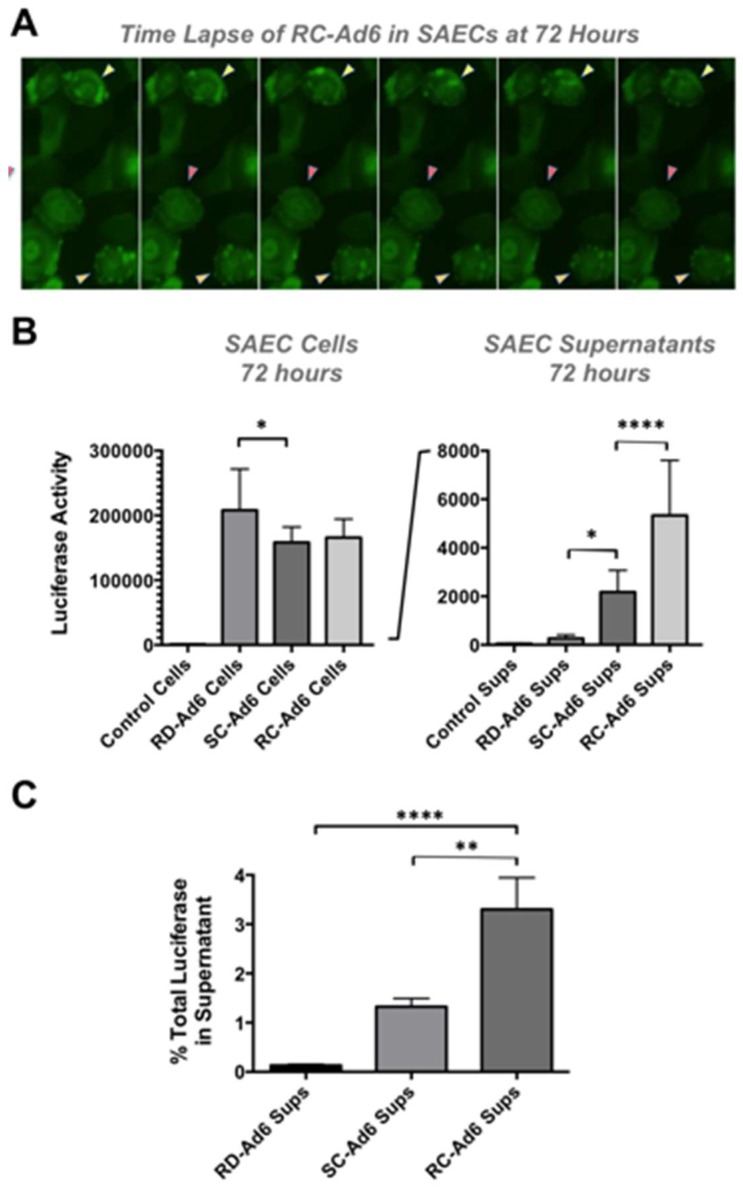
Cytotoxic effects of SC- and RC-Ad on cells and transgene protein localization. (**A**) RC-Ad infected cells were visualized by fluorescence microscopy in a time lapse taken every 10 s. Arrows indicate cells with active membrane blebs moving across their surfaces. Similar blebbing was observed in SC-Ad infected cells, albeit in fewer cells; (**B**) Luciferase activity in cell lysates (Cells) and cell supernatants (Sups) at 72 h; (**C**) Percent of luciferase activity in cell supernatants (Sups/(Cells + Sups)) at 72 h. * indicates *p* < 0.05, ** indicates *p* < 0.01 and **** indicates *p* < 0.0001 by ANOVA for the indicated groups.

First, SAEC cells were infected with 100 virus particles (vp) per cell of RD-, SC-, or RC-Ad6-GL, which all express the GFP-luciferase fusion protein under the control of the cytomegalovirus (CMV) promoter (Figure 1 and Figure 2). For these viruses, the CMV-GFP-luciferase transgene cassette was placed between the fiber and E4 region as a site distant from other regions that were deleted to change virus functionalities (i.e., away from E1 and pIIIA genes). At varied times after infection, the cells were visualized for GFP expression by microscopy and quantified by fluorescence measurements, and DNA was extracted from the cells and quantified by qPCR (Figure 2 and Figure 3). GFP fluorescence rose rapidly in SC- and RC-Ad infected SAECs, peaking within 24 h (Figure 2 and Figure 3A). As shown by microscopy, SC- and RC-Ad infected cells began dying over 36 to 48 h. RD-Ad GFP and luciferase expression remained lower than SC- and RC-Ad through 48 h, but then exceeded the two replicating vectors by 72 h, when cell death commenced in SC- and RC-Ad infected cells (Figure 3B and Figure 4B). Viral genomes in the RD-Ad-GL treated cells all remained low over 3 days after infection. SC- and RC-Ad amplified their genomes over 2 to 3 days with SC-Ad levels exceeding those of RC-Ad in these primary cells (Figure 3A).

As cells died, GFP fluorescence microscopy revealed active membrane blebbing reminiscent of apoptosis [11,12] in RC- and SC-Ad-infected cells (Figure 4A and [13]). In addition, by 72 h, significant amounts of GFP-Luciferase transgene protein were released into the media of the cell culture by SC- and RC-Ad when compared to RD-Ad infected cells (*p* < 0.05 and 0.0001, Figure 4B). Transgene protein release from the cells was significantly higher for RC-Ad than SC-Ad at 72 h (*p* < 0.0001), consistent with more rapid cell death induced by the RC-Ad vector. Notably, whether this is caused by necrosis or post-apoptotic necrosis in the cells is unclear.

Luciferase release into the supernatant was used as a surrogate marker of the breakdown of membrane integrity and release of cytoplasmic contents. 0.13%, 1.33%, and 3.30% of total luciferase was released into the supernatant at 72 h by RD-, SC-, and RC-Ad, respectively (Figure 4C). Luciferase release by RC-Ad was significantly higher than both RD- and SC-Ad (*p* < 0.0001 and 0.01, respectively. SC-Ad release was higher than RD-Ad, but was not significant. This suggests that RC-Ad kills primary human cells with somewhat accelerated kinetics than SC-Ad.

### 3.2. Activation of Interferon Stimulated Genes (ISG) by Ads

In vitro data in primary human lung cells show more rapid and robust transgene expression by SC- and RC-Ad than RD-Ad, with SC-Ad replication and expression significantly higher than RC-Ad. However, RD-Ad transgene expression catches up as SC- and RC-Ad infected cells die. While this could mean RD-Ad could be more potent than the DNA replicating vectors, this “catch up” in expression by RD-Ad is observed only in vitro under static conditions in the absence of an immune system. In contrast, RD-Ad expression is never higher than expression mediated by SC- and RC-Ad in vivo in hamsters [7].

In addition to this in vitro data, we previously showed in hamsters that SC-Ad drives markedly stronger systemic and mucosal immune responses than both RD- and RC-Ad after intranasal immunization [7]. While we expected SC-Ad to be better than RD-Ad, the results with RC-Ad were unexpected, since it should theoretically generate progeny virions for a second wave of infection of more cells to further amplify transgene expression and immune responses. However, the kinetics of luciferase expression is not extended in RC-Ad infected mice or hamsters, and we have seen no evidence for a second wave of RC-Ad infections [7,13].

These data suggest that RC-Ad may encounter some immunological barrier in vivo that SC-Ad does not, which suppresses its ability to spread and represses immune responses against transgenes. At the cellular level, both viruses amplify viral DNA and viral proteins similarly, but SC-Ad fails to package its DNA into virions and produces empty viral particles [6]. In contrast, RC-Ad does package its DNA and creates infectious progeny viruses. Therefore, the two vectors may provoke different intracellular or innate immune responses after infection that may affect their ability to drive transgene-directed immune responses in vivo.

One relevant cellular response to infection is activation of interferon stimulated genes (ISG) [14]. Ads evade many antiviral responses, but do induce ISGs by activating the cyclic GMP-AMP synthase (cGAS)/STING cascade [15,16]. cGas is a DNA sensor that induces STING, which activates antiviral responses via interferon (IFN) response factor 3 (IRF3). Phosphorylation of IRF3 induces transcription of ISGs, including IFN-β, ISG15, and ISG54 [15]. Notably, activation of the cGAS/STING pathway does not negatively affect the Ad life cycle [16]. However, differential activation of this pathway by RD-, SC-, and RC-Ad may influence immune responses against the transgenes that they carry.

To test whether the vectors differentially induce ISGs, THP-1 Lucia^tm^ ISG cells were infected with RD-, SC-, and RC-Ad6 viruses. THP1-Lucia™ ISG cells are human monocytes that express most cytosolic DNA sensors including cGAS/STING. These cells express secreted Lucia luciferase under the control of an IRF-inducible promoter and are unresponsive to NF-kB or AP-1 pathways. Unlike the firefly luciferase in the Ad6-GL viruses that uses luciferin as a substrate, Lucia uses coelenterazine, so its secreted activity can be discriminated from vector activity.

**Figure 5:**
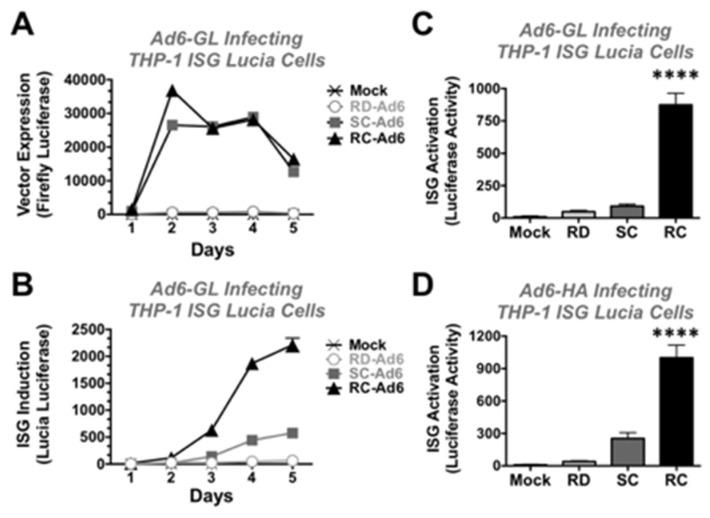
Interferon stimulated gene induction by Ad vectors. THP-1 ISG Lucia cells were infected at 10,000 vp/cell and firefly or Lucia luciferase activity was measure. (**A**) Vector-driven firefly luciferase expression after infection at 1000 vp/cell; (**B**) ISG-Lucia induction by on day 4; (**C**,**D**) Day 4 Lucia induction by vectors expressing GFP-Luciferase (**C**) or influenza hemagglutinin (HA) (**D**). **** indicates *p* < 0.0001 by ANOVA for RC-Ad when compared to Mock, RD, and SC-Ad groups.

THP-1 Lucia cells were infected at 1000 vp/cell with RD-, SC-, and RC-Ad6-GL and firefly and Lucia luciferase activity were measured over time (Figure 5). Similar to the results in the SAECs, SC- and RC-Ad6-GL amplified firefly luciferase expression within 2 days of infection significantly higher than RD-Ad in the THP-1 cells and this activity declined over time (Figure 5A).

On the other hand, ISG-Lucia expression gradually climbed over 5 days, with markedly higher ISG activation by RC-Ad than the other two vectors (Figure 5B). When this was repeated and measured at day 4, RD- and SC-Ad increased ISG-Lucia activity 5 and 10-fold, but this did not reach statistical significance compared to mock infected cells. In contrast, RC-Ad induced Lucia 97-fold (Figure 5C, *p* < 0.0001).

To validate this with non-firefly luciferase expressing vectors, RD-, SC-, and RC-Ad6 expressing influenza hemagglutinin (HA) were also tested in the THP-1 cells (Figure 5D). RD- and SC-Ad6-HA induced Lucia 4 and 28-fold, respectively, but were not significant. In contrast, RC-Ad6-HA induced ISG-Lucia 111-fold (*p* < 0.0001).

### 3.3. Adenovirus Luciferase Activity In Vivo in Mice

In vitro, RD-Ad transgene expression caught up to and exceeded SC- and RC-Ad expression by 72 h. While this could mean RD-Ad could be more potent than the DNA replicating vectors, this “catch up” in expression by RD-Ad is observed only in vitro under static conditions in the absence of an immune system. In contrast, RD-Ad expression is never higher than expression mediated by SC- and RC-Ad in vivo in hamsters [7].

**Figure 6:**
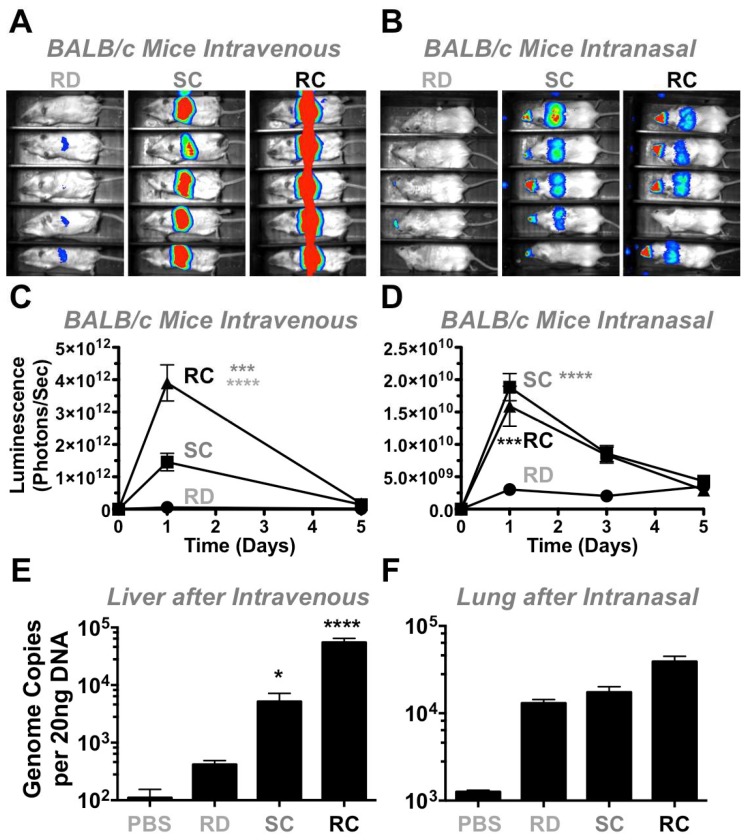
In vivo luciferase expression and genome replication in immunocompetent mice. Female BALB/c mice were injected with 3 × 10^10^ vp (IV) or 1 × 10^10^ vp (IN). (**A**,**B**) Mice were imaged with lumazone imager 24 h post-injection: intravenous 3 min 1 × 1 binning, 875–8750 grey values; intranasal 10 min 3 × 3 binning, 704–1200 grey values. Shown are representative white light images overlaid with luciferase expression in pseudo-color; (**C**,**D**) Quantification of luciferase images at days 1 and 5; (**E**,**F**) After 24 h, liver or lung samples were collected and total DNA was isolated. Viral genomes were quantified by qPCR. * *p* < 0.05. *** *p* < 0.001. **** *p* < 0.0001. Means ± SEM (standard error).

To test the vectors in vivo in a different animal model, 3 × 10^10^ vp of RD-, SC-, and RC-Ad6 were administered to immunocompetent female BALB/c mice by the intravenous route by tail vein and luciferase activity was measured one and five days later (Figure 6A,C). On day 1, there was higher luciferase expression in the livers of mice injected with SC-Ad6 than in animals injected with RD-Ad6, suggesting mouse liver cells support some level of Ad DNA replication (Figure 6A,C). For both vectors, luciferase expression declined to baseline by day 5.

To analyze an additional infection route and tissue, 10^10^ vp of each vector were administered intranasally in mice (Figure 6B,D,F) and luciferase activity was measured 1, 3, and 5 days after treatment. On days 1 and 3, there was significantly higher expression by both RC- and SC-Ad6 than RD-Ad6 in the nares and lungs of the animals, which decreased to baseline by day 5 (Figure 6B,D).

This trend is similar to the levels observed after intravenous injection, but these luciferase levels were 100-fold lower by the nasal route than were observed in the liver. Under these conditions, RC- and SC-Ad luciferase expression was only 5-fold higher than RD-Ad6 intranasally as compared to the 28-fold difference after intravenous injection, suggesting lung cells are less supportive of Ad DNA replication. For both infection routes, unlike observations in vitro, RD-Ad expression never exceeded expression by the replicating vectors in vivo, possibly due to the presence of an intact immune system.

### 3.4. Adenovirus Genome Replication In Vivo in Mice

BALB/c mice were injected intravenously and their livers were harvested one day later to quantify viral DNA replication in the tissue by qPCR (Figure 6E). These data indicated that there were 13- and 100-fold higher viral genome copies in the livers of SC- and RC-Ad6-treated mice than in RD-Ad6-treated animals. After intranasal infection, we were unable to isolate tissue and DNA from the nares of the animals where most luciferase expression was observed. Instead, we performed qPCR for viral DNA in the lungs of treated animals demonstrated only a 1.3-fold increase in genome copies of RC-Ad6 versus RD-Ad6 (Figure 6F), compared to the 13-fold increases observed in livers after intravenous injection. This suggests that mouse liver supports Ad6 DNA replication better than lung tissue.

### 3.5. Adenovirus Luciferase Expression In Vivo in Immunodeficient Mice

**Figure 7:**
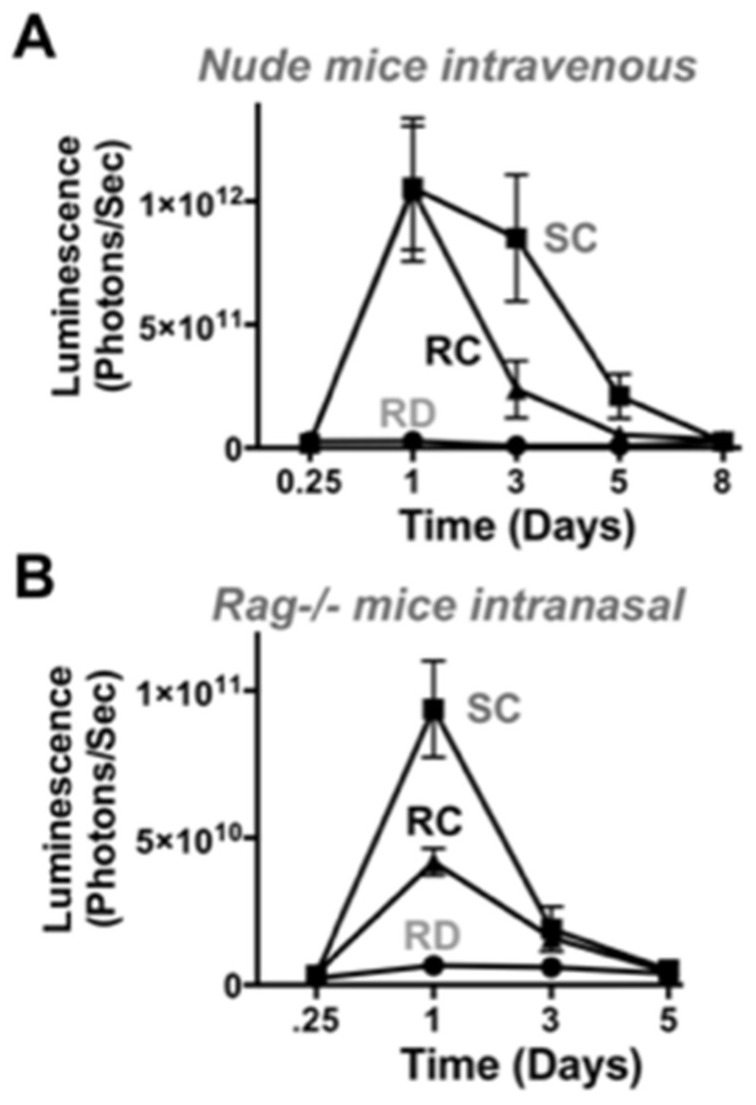
In vivo luciferase expression in immunodeficient mice. (**A**) Female nude mice were injected with 3 × 10^10^ vp (IV) and luciferase was imaged on the indicated days; (**B**) Female Rag-/- mice were administered 1 × 10^10^ vp by the intranasal route and luciferase imaging was performed.

To further determine the effect of the immune system on transgene expression, we measured luciferase expression in immunodeficient mice. When athymic nude mice were injected by the intravenous route, luciferase activity was equal to or higher in the SC-Ad group than in RC- or RD-Ad injected animals (Figure 7A). When immunodeficient Rag-/- mice were given the same vectors by the intranasal route, luciferase activity by SC-Ad was 2-fold higher than RC-Ad and both were markedly higher than luciferase produced by RD-Ad (Figure 7B). Despite the absence of a functional adaptive immune response, RD-Ad expression never exceeded expression by the replicating vectors in vivo, as was seen in vivo.

### 3.6. Discussion

In this study, we compared the ability of Ad vectors with different DNA and virion replication capabilities to replicate their DNA, produce transgene proteins, and induce immune responses and cell death in vitro and in vivo. We conducted these experiments to gain a better understanding of our previous results, which demonstrated a superior ability of SC-Ad6 to generate transgene-specific immune responses compared to RC-Ad6. RC- and SC-Ads replicate their DNA and transgenes to amplify protein expression. RC-Ad replicates DNA up to 10,000-fold and packages much of this DNA into virions. SC-Ad′s deletion of the pIIIa cement protein prevents this DNA from being packaged. Both RC- and SC-Ad also ultimately kill the infected cell which will effectively terminate antigen production. RD-Ad will not amplify genes or protein, but also will not by itself kill cells unless the transgene protein is toxic. This holds true in vitro. However, any cells infected by any of the vectors in vivo will ultimately be sacrificed by the innate or adaptive immune system unless they can become persistent infections. RD- and SC-Ad are engineered to prevent a second cycle of infection. In contrast, RC-Ad has the opportunity to infect a second set of cells, however, we have never observed this overtly even in permissive Syrian hamsters [7,13].

To examine how these vector differences affect protein expression and how they affect the cell they infected, Ad6 vectors expressing the GFP-luciferase transgene protein were first compared in vitro in primary human lung epithelial cells. SC- and RC-Ad6 both replicated their DNA, but SC-Ad amplification was higher. Both also amplified GFP production over 48 h above that of RD-Ad. By 72 h, the two replicating vectors begin killing the cells, with RC-Ad cell death occurring somewhat faster than SC-Ad. When luciferase with its activity was measured at this time in cells, RD-Ad expression was somewhat higher. However, when cell supernatants were examined, luciferase protein was being released from the cells secondary to cell death induced by the replicating vectors. This expression, cell death, and antigen disposal likely impact how the immune system detects antigens expressed by the different vectors in vivo.

In addition to antigen release from the cells, the viruses have core differences in their viral life cycle that could induce innate immune responses. Notably, SC- and RC-Ad replicate DNA, but RD-Ad does not. RC-Ad packages this amplified DNA, but SC-Ad does not. Therefore, it was possible that this DNA (or other viral components) could serve as “danger signals” to the immune system during infection by these different vectors. To test this, THP-1 monocytes carrying an ISG sensing reporter gene were infected with the vectors. Under these conditions, SC- and RC-Ad again amplified transgene protein over RD-Ad, but in these cells RD-Ad never caught up or exceeded the replicating vectors. Transgene protein plateaued within 2 days, but ISG activation climbed over 4 to 5 days. When Ads expressing GFP-luciferase or influenza HA were compared at day 4, ISG stimulation by RC-Ad was significantly higher than SC- or RD-Ad and SC-Ad was somewhat higher than RD-Ad. This 100-fold higher ISG activation by RC-Ad, and not SC-Ad, may trigger stronger “danger signal” activation in vivo which may dampen the ability of RC-Ad to drive immune responses compared to SC-Ad. This may also explain the lack of an obvious second cycle of infection by RC-Ad in hamsters as the innate immune response suppresses viral expansion in vivo.

Mice are excellent models to test vectors, since they are inbred and have a wide spectrum of immunologic reagents. Unfortunately, they have been shown to not support the full progeny virus production cycle [17]. Mice and certain mouse cell lines have previously been shown to support low levels of replication [18]. This work was focused on evaluating genome replication and transgene expression in the context of an immune system, regardless of the ability to replicate functional progeny virions.

When the vectors were tested in vivo in mice and found that vector genome replication capacity was dependent on route of delivery. When the viruses were delivered intravenously, luciferase expression from SC-Ad6 was 13 times higher than RD-Ad6. This corresponded to 28-fold increase in Ad6 genome copies by SC-Ad6 when compared to RD-Ad6. In contrast, when the same viruses were delivered intranasally, genome replication and transgene expression was dampened. By this route, luciferase expression by SC-Ad6 was only 5 times higher and its genome copies were only 1.3 times higher than the levels that were generated by RD-Ad6. This suggests that this route does not fully manifest viral genome replication and transgene amplification as well as the intravenous route in mice.

In mice that do not support the production of infectious progeny viruses, SC- and RC-Ad should be equal. After intravenous injection, RC-Ad was better than SC-Ad and both expressed luciferase more strongly than RD-Ad at all time points. After intranasal administration, SC-Ad was stronger than RC-Ad and both were again stronger than RD-Ad. Unlike the in vitro situation, RD-Ad expression never caught up or exceeded SC- and RC-Ad in either tissue.

These different responses after intranasal and intravenous vector delivery may be related to the differing immunological balances that are present in the different tissues. Given the wet and thin structure of the mucosa of the respiratory tract, it is a front line for pathogen entry into the body and has evolved to efficiently detect and quickly repel incoming pathogens. In contrast, the primary function of the liver is in digestion [19]. The liver is constantly bathed in foreign antigens generated during digestion by the gastrointestinal tract. The liver must absorb foreign digestive antigens without provoking inflammatory immune responses. It must also detect blood-borne pathogens. The liver therefore strikes somewhat of a more immunologically tolerant balance than mucosal surfaces [19]. It is therefore possible that RC-Ad is better than SC-Ad in the liver by virtue of its somewhat better DNA replication, and there may be lower responses to RC-Ad′s stronger ISG induction. Conversely, SC-Ad may express better than RC-Ad in more reactive respiratory tissues by virtue of driving reduced danger signaling with its lower ISG induction.

In immunodeficient nude and RAG-/- mice, SC-Ad expressed transgenes at higher levels than RC-Ad by both routes. SC-Ad was better than RC-Ad by the intranasal route in immunocompetent and in immunodeficient RAG knock-out mice. In contrast, RC-Ad was better in the liver in immunocompetent mice, but worse in immunodeficient nude mice. While nude mice are more known for their defects in T cell responses, these T cell defects perturb B cell maturation and resulting immunoglobulin levels and increase the numbers of activated macrophages in the animals [20,21,22]. After intravenous injection, Ad is rapidly bound by natural IgM antibodies, followed by complement fixation, and targeting to liver and spleen macrophages [21,22]. It is therefore possible that the more inflammatory RC-Ad vector may be more readily detected in nude mice than in normal mice resulting in reduced expression after intravenous injection. It is also possible that the stronger reaction against RC-Ad may reduce this virus′ level of DNA replication and/or gene expression to a greater degree in immunodeficient mice. If so, this may be detected by monitoring DNA replication in the lungs and liver after intranasal and intravenous administration. This would ideally be performed head to head in BALB/c vs. BALB/c nude mice or congenic RAG-/- mice.

In hamsters (that are reported to support the full Ad lifecycle [17]), RC-Ad should express genes longer than SC-Ad and drive more robust immune responses if it is able to proceed to second and third cycles of infection occur. However, we have never observed extended expression by RC-Ad over SC-Ad in hamsters in vivo [7]. In actuality, SC-Ad drives markedly stronger and more persistent immune responses against transgene proteins than RC-Ad in hamsters [7]. Notably, both vectors generate equal anti-Ad responses despite the difference in responses against transgene antigens. This is consistent with a lack of a second wave of RC-Ad infection and the absence of additional Ad protein production. This also points towards differences in responses to the transgene protein or the vector driven by the two vectors perhaps via the stronger ISG and antiviral responses against RC-Ad than SC-Ad than in vivo. Similar effects have been observed when testing single-cycle and RC flavivirus vaccines [8] where single-cycle vaccines drive more robust responses than RC vaccines. This suggests that there is a common immunological effect in different vaccine systems.

In most current E1-deleted RD-Ad vectors, transgene cassettes are inserted in place of the E1 gene. In our vector comparisons, transgene cassettes were placed between the fiber and E4 region in the Ad to avoid moving the cassette within the virus and creating another variable when E1 and pIIIA were deleted. While this controlled this variable, this site is likely not the most ideal for transgene cassettes. Placing cassettes in the E1 region places the transcription unit adjacent to the more open region of the viral genome near the packaging signal and inverted terminal repeats. This more open DNA region enhances gene expression in cis. In contrast, the fiber-E4 insertion site is at a position where the 3′ ends of the major late transcripts and the E4 mRNAs both converge and expression is lower than in the E1 site. Therefore, a current RD-Ad vector with its genes in E1 may perform better than the E1 deleted vectors used here with their genes in the neutral fiber-E4 site.

Now that we have established the utility of SC-Ad for transgene and vaccine expression, we are testing alternate sites to amplify efficacy and improve safety. For example, we have previously inserted transgenes like GFP-luciferase and FX-single-chain antibodies in between E1A and B in RC-Ads in this more open region of the viral genome [23,24]. We are therefore testing this and E1 adjacent sites for their ability to drive more robust transgene expression than the fiber-E4 site.

These data provide insight into the differing biologies of RD-, SC-, and RC-Ad vaccines, and further support the development of SC-Ad vectors as more robust platforms for gene-based vaccination and transgene expression for cancer and other therapies.

## Abbreviations

The following abbreviations are used in this manuscript:

Ad	adenovirus
GL	green fluorescent protein-luciferase fusion protein
HA	hemagglutinin
ISG	interferon-stimulated genes
RD-Ad	replication-defective adenovirus
RC-Ad	replication-competent adenovirus
SC-Ad	single-cycle adenovirus
